# Impact of heat processing on the nutritional content of *Gryllus bimaculatus* (black cricket)

**DOI:** 10.1111/nbu.12374

**Published:** 2019-04-30

**Authors:** D. Dobermann, L. M. Field, L. V. Michaelson

**Affiliations:** ^1^ Rothamsted Research Harpenden UK; ^2^ University of Nottingham Nottingham UK

**Keywords:** black crickets, edible insects, heat processing, nutrition

## Abstract

Insects are increasingly suggested as a potential novel solution to global nutrition challenges. However, limited research is available on the impact of processing methods on the nutritional content of edible insects. This trial examines the effect of heat processing on the nutritional profile of the black cricket, *Gryllus bimaculatus*. Adult black crickets were killed by freezing and then dried at either a low (45°C) or high (120°C) temperature followed by nutritional analysis of protein and micronutrient content. An additional set of samples was either freeze‐dried or dried at 32, 45, 72 or 120°C followed by nutritional analysis of lipid content. Analysis showed that percentage protein content was significantly higher in crickets dried at 45°C, a difference of roughly 1% of the total weight. Similarly, calcium content was also significantly higher in crickets dried at 45°C, although no other measured micronutrients were affected. Additionally, the fatty acid content was significantly influenced by higher temperature processing. Freeze‐drying black crickets conserved significantly more of the long‐chain polyunsaturated fatty acids than drying at 120°C. Insects hold potential as a source of essential nutrients and fatty acids; however, consideration must be given to heat processing at high temperatures as this may affect the nutritional profile.

## Introduction

One of the suggested solutions to malnutrition globally is an increase in the use of edible insects, specifically to combat micronutrient deficiencies (van Huis *et al*. 2013; Nadeau *et al*. 2014). Insects are a valuable source of protein and contain vital lipids, micronutrients and amino acids (Bukkens 1997; Ramos‐Elorduy *et al*. 1997; Rumpold & Schlüter 2013) and are a regular part of the traditional diet for at least 2 billion people in 113 countries (van Huis *et al*. 2013; Rumpold & Schlüter 2013). Thus, for many people, the potential use of insects to treat malnutrition could be a cheap solution that is in line with existing food habits and preferences (Horton 2006; Moreira‐Araújo *et al*. 2008).

A small number of trials have formulated insect‐based intervention foods and analysed these for nutritional properties, storage capability and/or sensory acceptability. One such trial used termites (species not specified) in combination with *Rastrineobola argentea* (silver cyprinid fish) and found the termite‐supplemented food provided higher levels of energy, protein, fat and zinc than the same complementary food without termites; it was also found to have a shelf life of 6 months before harmful microbial activity occurred (Kinyuru *et al*. 2015). Similarly, cookies made with 10% flour made from *Rhychophorus phoenicis* (palm weevil) larvae had 85% more protein and a higher energy value than those made with standard wheat flour (Adeboye *et al*. 2016). In another study, termite‐based foods were just as acceptable to infants and mothers as corn‐soy blend foods (Konyole *et al*. 2012) and there were no significant differences in aroma and taste acceptability scores of wheat flour buns enriched with between 0% and 20% of termite flour (Kinyuru *et al*. 2009). Another trial developed a caterpillar‐based cereal product for children, comprising dried caterpillars (species not specified), ground corn, sugar, salt and palm oil. Thirty gram portions of the product, which met the World Health Organization nutritional requirement guidelines for protein and fat levels of a complementary food for 6–11 month‐old infants, were provided to 20 mother–child dyads for a week and the acceptability assessed. Overall, the product was found to be biologically safe and highly acceptable to the mothers and infants (Bauserman *et al*. 2015a). The trial was extended to 175 6 month‐old infants, who were either provided with the caterpillar‐based cereal product as a complementary food or not on a daily basis for 12 months, after which anthropometric and biological samples were collected. No difference in stunting prevalence between the two test groups was found; however, the group fed on the caterpillar‐based cereal product showed fewer cases of anaemia, suggesting it offered micronutrient benefits (Bauserman *et al*. 2015b). To date, this is the only trial to have directly utilised an insect‐based food in a nutrition intervention.

One potential limiting factor in the use of insects as a nutritional intervention food is the high degree of variability seen in the nutritional content of insects, even within the same species (Bukkens 1997; Ramos‐Elorduy *et al*. 2002). Reviews of the nutritional composition of edible insects have identified clear within‐species variation in the proportions of macronutrients and micronutrients (Payne *et al*. 2016; Dobermann *et al*. 2017). For example, the reported standard deviation of the manganese content of *Acheta domesticus* (house cricket), 1.66 mg ±0.906, exceeds 50% of the mean (Payne *et al*. 2016) and for *Tenebrio molitor* (yellow mealworm) larval calcium content has been reported to range from 13 to 472 mg/100 g (Nowak *et al*. 2016).

It is well established that food processing, particularly using heat, can cause nutritional degradation of many foods (Lund 1988) and this is also true for insects. For example, the preparation of *Imbrasia epimethea* (African moth) via evisceration and cooking, or drying and evisceration, reduces the amount of carbohydrates and heating reduces the amount of monosaturated fatty acids (Lautenschläger *et al*. 2017). A similar trial using *Imbrasia belina* (mopane worm) larvae found that the cooking method affected the ash, calcium, phosphorous, zinc, manganese and crude protein content (Madibela *et al*. 2007). However, in this trial all samples, regardless of initial processing, were ultimately dried at 105°C for 24 hours prior to processing. This step introduces a significant confounding variable, making it difficult to establish if the nutritional changes were due to the first processing steps or the subsequent heating. There have been no trials where insects were dried at multiple temperatures and then analysed for the impact of drying.

These findings highlight that processing may influence the nutritional content of insects. This is important because insects bred for food are currently not processed in a uniform manner. The trial reported here examined the effect of heat processing on the nutritional profile of the insect, *Gryllus bimaculatus* (black cricket). This species was selected because of the wide popularity of crickets both as a traditional food and as a ‘gateway’ insect commonly eaten in the form of flour or protein bars in countries which do not presently consume insects.

## Materials and methods

This study was designed to examine the impact of heat processing on the nutritional content of the edible black cricket. Outcome measures were protein content, micronutrient content (arsenic, calcium, cadmium, cobalt, iron, potassium, magnesium, manganese, sodium, lead, selenium and zinc) and fatty acid profile [total fatty acids and the individual fatty acids: palmitic (16:0), stearic (18:0), oleic (18:1), linoleic (18:2) and α‐linolenic acid (18:3)].

### *Gryllus bimaculatus* (black cricket)

Adult black crickets were sourced from Monkfield Nutrition Ltd, Royston, UK, where they had been reared on a standard commercial chicken feed diet for 4–5 weeks.

### Analyses

All black crickets were killed by freezing at −20°C for 24 hours. Following this, one set of black crickets was dried at either 45 or 120°C for 36 hours for protein and micronutrient analysis. For fatty acid analysis, black crickets were dried at either 32, 45, 75 or 120°C for 36 hours, and one set of black crickets was freeze‐dried. Black crickets were dried over a larger range of temperatures for fatty acid analysis as fatty acids are known to be particularly sensitive to heat processing (Lautenschläger *et al*. 2017).

#### Protein content

Three hundred milligrams of each black cricket sample was placed in the autosampler of a Leco TruMac Combustion Analyser (Dumas method) to determine total nitrogen and carbon, using five replicates per temperature. Protein content was determined by multiplying the nitrogen content by 6.25 (Jones 1941; Sosulski & Imafidon 1990). Although recent research suggests that the 6.25 nitrogen to protein conversion factor, which has been standard until now, potentially overestimates protein content (Janssen *et al*. 2017), any overestimation would affect all of the treatments equally and therefore the comparisons between treatments remain valid. Protein content is expressed as a percentage of the total weight of the insects.

#### Micronutrient content

Five hundred milligrams of each black cricket sample was placed in 25 ml graduated digestion test tubes, using five replicates per temperature, with one sample out of every three repeated to check analysis accuracy. Two wheat flour samples were included as a certified standard and two blank control tubes to establish any potential contamination in the solvents. Samples were pre‐digested in 5 ml of 15:85 nitric:perchloric acid (HNO_3_:HClO_4_) for 5 hours then heated overnight to 175°C to evaporate the acid. Five millilitres of 25% HNO_3_ was added, and the tubes heated to 80°C for 60 minutes. Samples were made up to a final volume of 25 ml with ultra‐pure water, decanted and analysed using Optima Inductively Coupled Plasma – Optical Emission Spectrometer (ICP‐OES) (Zhao *et al*. 1994). All nitric acid was Aristar grade unless otherwise stated.

#### Fatty acid content

Twenty micrograms of each black cricket sample was analysed, using five replicates per temperature. Samples were placed in 4.5 ml glass vials, and 5 μl methyl heptadecanoate (C17:0) was added as an internal standard. Glass vials and pipettes were used throughout the procedures. Extraction/methylation mix (1.5 ml), comprising 85% MeOH, 10% HCl, 5% 2‐2, dimethoxypropane, was added, and the vials were capped and heated to 80–85°C for 1 hour. Vials were then cooled, and 1 ml of 1% NaCl was added. The samples were mixed and the fatty acids extracted into 1 ml hexane. Following methylation, the hexane fraction was concentrated under nitrogen and resuspended in 300 μl solvent prior to injection of 1 μl of a 1 in 10 dilution, onto the gas chromatography column. Methyl ester derivatives of the total fatty acids extracted were analysed by gas chromatography (Agilent 7890A) using an Agilent DB‐225 column (size: 30 m × 0.32 mm × 0.3 μm). The inlet and detector temperature were set to 250°C, and 2 μl of each sample was analysed using splitless injection and a constant flow rate of 2 ml/min. Chromatograms were analysed using Agilent ChemStation software. The retention time and identity of each fatty acid methyl ester (FAME) peak were calibrated using the FAME mix rapeseed oil standard (Supelco™, Sigma‐Aldrich Ltd., Poole, UK).

#### Data analyses

Data were analysed using IBM SPSS Statistics 22 (International Business Machines Corporation (IBM), Armonk, NY, USA) and Microsoft Excel. Differences between temperature groups (45 or 120°C) for protein and micronutrient content were analysed using independent samples t‐tests. Differences between temperature groups (32, 45, 75, 120°C or freeze‐dried) for fatty acids were analysed using one‐way between‐subjects analysis of variance (ANOVA). ANOVA with Welch's *F*‐test was used when Levene's test of homogeneity of variance was violated. Scheffe's *post hoc* tests were used to explore differences between specific temperatures. Significance was set at the *P *>* *0.05 level. Where appropriate, standard errors or standard deviations are reported.

## Results

### Protein content

**Figure 1:**
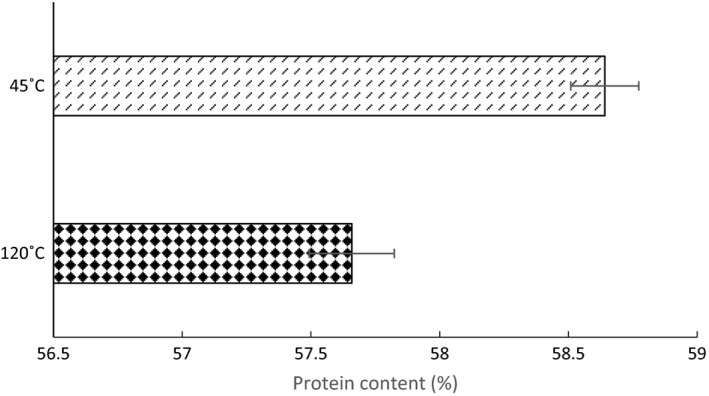
Average protein content (% of total weight) in black crickets dried at 120°C (checked bar) and 45°C (lined bar) with ± standard deviation.

There was a significant difference in protein content between black crickets dried at 45 and 120°C [*t* (8) = −10.467, *P *<* *0.0001]. Figure 1 displays the average protein content in black crickets dried at the two temperatures. Those dried at the lower temperature had around 1% higher protein content than those dried at the higher temperature.

### Micronutrient content

**Figure 2:**
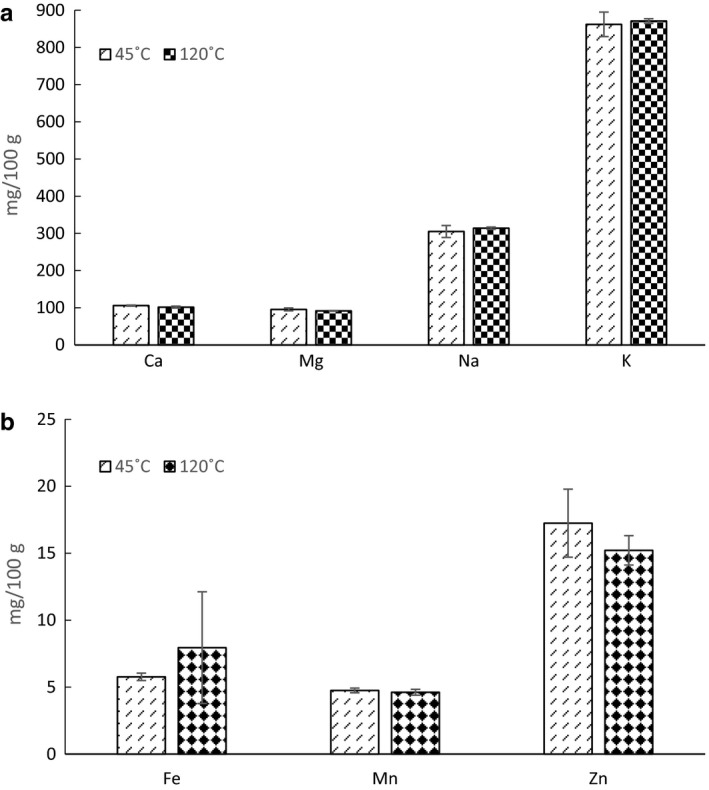
(a, b) Average content of selected micronutrients (mg/100 g) in black crickets dried at 45 and 120°C with +/− standard deviation. Ca, calcium; Mg; magnesium; Na, sodium; K, potassium; Fe, iron; Mn, manganese; Zn, zinc.

Arsenic, cadmium, cobalt, lead and selenium were not present within ICP‐OES detection limits. Of the other micronutrients displayed in Figure 2, only calcium content showed a significant difference between the two temperatures [*t* (8) = −3.181, *P *=* *0.013] with black crickets dried at the 45°C containing the most calcium. There were no significant differences between the high and low processing temperature for magnesium [*t* (8) = −2.291, *P *=* *0.051], sodium [*t* (8) = 1.201, *P *=* *0.264], potassium [*t* (8) = 0.583, *P *=* *0.576], iron [*t* (8) = 1.165, *P *=* *0.278], manganese [*t* (8) = −1.106, *P *=* *0.301] or zinc [*t* (8) = −1.645, *P *=* *0.139].

### Fatty acid content

**Figure 3:**
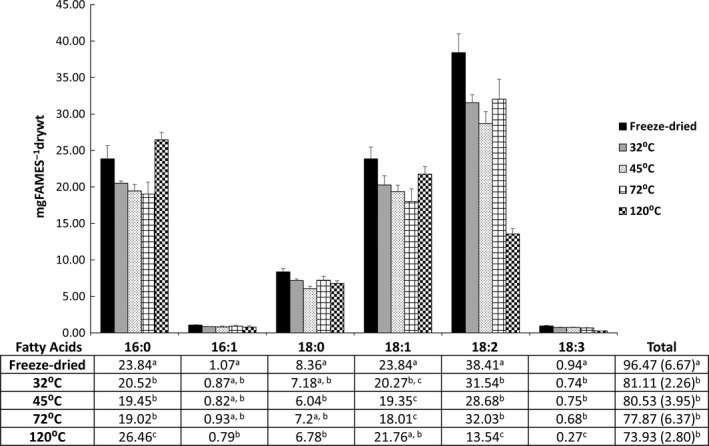
Average amount [mg of fatty acid methyl esters (FAMEs) per g of dry weight] of specific fatty acids in black cricket processed at various temperatures, *n* = 5. Error bars ± standard deviation (SD). Different letters within a column indicate significant differences based on Scheffe's *post hoc* tests, *P *<* *0.05. Palmitic acid (16:0), stearic acid (18:0), oleic acid (18:1), linoleic acid (18:2), α‐linolenic acid (18:3).

There was a significant main effect of heat treatment on the total fatty acid content [*F* (4, 20) = 16.21, P < 0.0001], with total fatty acid content decreasing as temperature increased (see Fig. 3). There was also an overall significant effect of heat treatment on the amount of palmitic acid [16:0; Welch's *F* (4, 12.55) = 38.13, *P *<* *0.0001], stearic acid [18:0; Welch's *F* (4, 12.77) = 12.29, *P *<* *0.0001], oleic acid [18:1; Welch's *F* (4, 11.86) = 10.96, *P *<* *0.001], linoleic acid [18:2; Welch's *F* (4, 10.26) = 365.4, *P *<* *0.0001] and α‐linolenic acid [18:3; Welch's *F* (4, 10.28) = 394.39, *P *<* *0.0001] found in the black crickets, Figure 3.

*Post hoc* tests (see Fig. 3) showed that freeze‐drying the black crickets conserved significantly more of both the longer chain fatty acids [linoleic acid (18:2) and α‐linolenic acid (18:3)] and those with more than one desaturation than any of the other drying temperatures (*P *<* *0.05). Although, interestingly, there was no significant effect on the amount of oleic acid (18:1) between black crickets which were freeze‐dried and those which were dried at 120°C (*P *>* *0.05). Drying at 120°C resulted in significantly higher amounts of palmitic acid (16:0) and lower amounts of linoleic acid (18:2) and α‐linolenic acid (18:3) than any of the other drying temperatures (*P *<* *0.05). There was no difference in fatty acid content between black crickets dried at 32, 45 or 72°C (all *P *>* *0.05).

## Discussion

To date, only a limited number of studies have examined the impact of processing temperature on the nutritional profile of edible insects, and previous studies have done this predominantly within the context of how the insects are traditionally prepared rather than systematically (Mutungi *et al*. 2017). This study examined the impact of various drying temperatures on the nutritional content of black crickets and found that while some effect is seen on protein and micronutrient content, the most marked change is seen in the fatty acid profile, with black crickets dried at higher temperatures showing lower levels of polyunsaturated fatty acids (PUFAs).

The finding that the protein content was slightly higher in the black crickets which were dried at 45°C, compared to 120°C, is in direct agreement with a previous paper which found that oven dried housefly larvae had lower protein levels than sun‐dried larvae (Aniebo & Owen 2010).

The finding that temperature treatment predominantly does not affect the micronutrient components of black crickets is in line with previous studies (Madibela *et al*. 2007; Mutungi *et al*. 2017), although neither of these studies compared a distinct set of temperatures but rather distinct processing methods such as boiling versus frying. The finding that micronutrients such as iron, potassium and zinc in black crickets are largely not affected by processing temperature is important as it suggests that insects can be processed into foods such as buns, biscuits, flour and porridge without reducing their micronutrient value.

The results of the present study show that at 120°C there is a sharp drop in the linoleic acid content of the crickets. This is in line with findings that PUFAs are sensitive to oxidation especially in combination with increasing temperatures above 60°C (Jacobsen & Let 2008). Further, it is possible that the PUFAs originally present in the black crickets were broken down via oxidation into the shorter chained palmitic acid (16:0) accounting for the increasing 16:0 content seen at higher drying temperatures. Black crickets dried at 32, 45, 72 or 120°C showed small differences in total fatty acid content, although their content was markedly lower than black crickets which were freeze‐dried. Given the caloric value of fats (Youdim 2016), the implications of this finding are that crickets dried using heat may be of slightly lower caloric value than freeze‐dried crickets.

Although fatty acids are often not considered in malnutrition, their role particularly in children's development should not be disregarded. The brain requires PUFAs for optimal growth and development, specifically eicosatetraenoic acid (20:4) and docosahexaenoic acid (22:6), which are synthesised from their respective essential fatty acid (EFA) precursors 18:2 (linoleic acid) and 18:3 (α‐linolenic acid) (Wainwright 1992). Essential fatty acid deficits have been identified in malnourished children and have been linked to a number of clinical problems such as increased risk of infection, impaired wound healing, fatty liver and psychomotor changes coupled with growth retardation (Aaes‐Jørgensen 1977; Hansen *et al*. 1963; Koletzko *et al*. 1986; Rivers & Frankel 1981).

The European Food Safety Authority (EFSA 2009) has proposed food reference intake values of 2 g/day for α‐linolenic acid and 10 g/day for linoleic acid for food labeling purposes. In the present trial, samples which were dried at 120°C had, on average, <15 mg of linoleic acid per gram of dry cricket, while all other drying temperatures had approximately twice as much linoleic acid. Similarly, the samples dried at 120°C had <0.3 mg of α‐linolenic acid, while other temperatures had closer to 1 mg per gram of dry crickets. Although these differences are relatively small, they may make a difference within diets when foods made of black crickets are consumed in sufficient amounts on a regular basis and this means that consideration should be given to the processing of insect‐based foods, particularly those aimed at children.

It is important to note that if heating during processing is kept below 120°C, microbial contamination can still be controlled by immersing in boiling water for 120 seconds prior to further processing. Previous work has also demonstrated that a short heat step is sufficient to reduce microbial load to safe levels (Klunder *et al*. 2012). The drying temperature made a significant difference in the fatty acid profiles of the crickets, and this is likely due to the extended nature of the heat exposure as drying occurs over several hours or days. At present, most insects consumed in rural developing communities are wild harvested and cooked directly or sun‐dried at temperatures that would not exceed approximately 50°C, meaning current practices in regions where insects are used as food may already preserve the fatty acid profile of insects making them more nutritionally valuable as a food source.

## Limitations and future directions

There are two key limitations to this study. First, because minimal effects of temperature processing on the protein and micronutrient content of the black cricket were anticipated, only two temperatures were tested. However, the present study suggests that protein and perhaps some micronutrients in black crickets are sensitive to heat treatment, and a wider range of temperatures would have been useful in elucidating at which temperature the most significant changes occur. Future research should explore the effects of a wider range of temperatures. Second, there is some evidence to suggest the gut contents of insects have an impact on their nutrient content (Mutungi *et al*. 2017) and this was not controlled for in the study. While the crickets had all been given free access to food prior to being dispatched, it is possible that some of the crickets had fuller guts than others. This could account for the significant group differences observed in calcium content, as the chicken feed used to rear the crickets is high in calcium. Future trials could control for this ‘gut load’ effect by degutting the insects or introducing a fasting period to ensure the digestive systems of the insects are empty.

Lastly, as it has been shown in this trial that fatty acids in black crickets are sensitive to processing temperature, a future trial should establish which temperature(s) retains the most desirable fatty acid profile. Establishing at which temperature changes in fatty acid content occur is particularly important for food manufacturers who need to balance nutritional quality with microbial safety; future recommendations on the processing of black crickets should take both nutrition and safety considerations into account (Rumpold & Schlüter 2013).

As edible insects are increasingly considered for nutrition interventions, it is important that researchers and food manufacturers are aware that heat processing has an impact on the nutrients they deliver as well as their safety. Insects have the potential to be a good source of essential nutrients and fatty acids, but only if processed correctly.

## Conflict of interest

The authors certify that they have no affiliations with, or involvement in, any organisation or entity with any financial interest or non‐financial interest in the subject matter or matters discussed in this manuscript.
